# rhKGF-2 Attenuates Smoke Inhalation Lung Injury of Rats *via* Activating PI3K/Akt/Nrf2 and Repressing FoxO1-NLRP3 Inflammasome

**DOI:** 10.3389/fphar.2021.641308

**Published:** 2021-07-22

**Authors:** Zhonghua Fu, Zhengying Jiang, Guanghua Guo, Xincheng Liao, Mingzhuo Liu, Zhenfang Xiong

**Affiliations:** ^1^ Department of Burn, The First Affiliated Hospital of Nanchang University, Nanchang, China; ^2^ Department of Pathology, The First Affiliated Hospital of Nanchang University, Nanchang, China

**Keywords:** smoke inhalation injury, rhKGF-2, inflammation, PI3K, FoxO1, NLRP3

## Abstract

Smoke inhalation injury is an acute pathological change caused by thermal stimulation or toxic substance absorption through respiratory epithelial cells. This study aims to probe the protective effect and mechanism of recombinant human keratinocyte growth factor 2 (rhKGF-2) against smoke inhalation-induced lung injury (SILI) in rats. The SILI was induced in rats using a smoke exposure model, which were then treated with rhKGF-2. The rat blood was collected for blood-gas analysis, and the levels of inflammatory factors and oxidative stress markers in the plasma were measured. The rat lung tissues were collected. The pathological changes and cell apoptosis were determined by hematoxylin-eosin (HE) staining and TdT-mediated dUTP nick end labeling (TUNEL) assay, and the PI3K/Akt/Nrf2/HO-1/NQO1, and FoxO1-NLRP3 inflammasome expression were verified by western blot (WB). Both of the human alveolar epithelial cell (HPAEpiC) and primary rat alveolar epithelial cell were exposed to lipopolysaccharide (LPS) for making *in-vitro* alveolar epithelial cell injury model. After treatment with rhKGF-2, GSK2126458 (PI3K inhibitor) and AS1842856 (FoxO1 inhibitor), the cell viability, apoptosis, inflammation, oxidative stress, reactive oxygen species (ROS), PI3K/Akt/Nrf2, HO-1/NQO1, and FoxO1-NLRP3 in HPAEpiC and primary rat alveolar epithelial cell were examined. The data suggested that rhKGF-2 reduced LPS-induced HPAEpiC cell and primary rat alveolar epithelial cell apoptosis and the expression of inflammatory factors and oxidative stress factors. Moreover, rhKGF-2 improved the blood gas and alleviated SILI-induced lung histopathological injury *in vivo via* repressing inflammation, NLRP3 inflammasome activation and oxidative stress. Mechanistically, rhKGF-2 activated PI3K/Akt pathway, enhanced Nrf2/HO-1/NQO1 expression, and attenuated FoxO1-NLRP3 inflammasome both *in vitro* and *in vivo*. However, pharmaceutical inhibition of PI3K/Akt pathway attenuated rhKGF-2-mediated protective effects against SILI, while suppressing FoxO1 promoted rhKGF-2-mediated protective effects. Taken together, this study demonstrated that rhKGF-2 mitigated SILI by regulating the PI3K/Akt/Nrf2 pathway and the FoxO1-NLRP3 axis, which provides new reference in treating SILI.

## Introduction

Smoke inhalation-induced lung injury (SILI) is a kind of thermal and chemical injury caused by high-temperature gases or harmful chemicals acting on the respiratory tract. It mostly occurs among fire victims or firefighters and is one of the main causes of fire-related deaths. According to recent statistics, the mortality rate of burn-associated inhalation injury reaches to 30% (Colohan, 2010). In terms of the mechanism, the overheated steam burning, the stimulation of incomplete combustion products, the airway obstruction by toxic particles, and the consequent inflammation are the main pathological mechanisms of SILI, which eventually lead to pulmonary edema, fibrosis, and aggravate hypoxia (Guo et al., 2019). The pathophysiology of SILI is complex, and it progresses rapidly. In addition, there is a lack of safe and effective drugs clinically. Therefore, this paper discussed the function of recombinant human keratinocyte growth factor 2 (rhKGF-2) in SILI and its potential as therapeutic drugs.

Keratinocyte growth factor 2 (KGF-2), also known as fibroblast growth factor 10 (FGF-10), is a soluble peptide secreted by fibroblasts and endothelial cells. It has about 30–60% amino acid sequence homology with the FGF family and acts on epithelial cells mainly by activating high-affinity transmembrane FGF-2 IIIb receptor (Yamasaki et al., 1996). Studies over the years have shown that KGF-2 contributes to lung development, injury, and repair (Danopoulos et al., 2019). For example, Chao et al. found that FGF-10 deficiency affects the formation of type II alveolar epithelial cells, which leads to congenital lung defects and weakens the lung’s ability to cope with hyperoxic injury (Chao et al., 2017). In addition, Gupte et al. showed that FGF-10 overexpression dampens bleomycin-induced pulmonary inflammation and fibrosis in mice and prolongs survival (Gupte et al., 2009). There is no research showing whether KGF-2 plays a role in SILI.

KGF-2 impacts disease progression alone or by regulating the downstream pathways. For instance, Chen et al. found that FGF10 exerts an anti-inflammatory and anti-apoptotic role by activating FGFR2/PI3K/Akt and hampering TLR4/NF-κB, thus improving post-injury repair of the central nervous system (Chen et al., 2017). This suggests that the regulation of KGF-2 on PI3K/Akt may be a potential target against inflammatory response. Protein kinase B (Akt) is the most easily activated effector of the phosphatidylinositol-3 kinase (PI3K) downstream. The close coupling of the two is essential for lung injury. Moreover, it has been confirmed that the PI3K/AKT pathway prevents high oxygen-induced lung injury by up-regulating Nrf2. Interestingly, this regulation is selective, and PI3K/Akt can maintain lung homeostasis without Nrf2 (Reddy et al., 2015). Nuclear factor-erythroid 2 related factor 2 (Nrf2) is the main regulator of the antioxidant response element (ARE)-mediated cytoprotective protein expression. Emerging studies have shown that Nrf2 activation prevents inflammation and oxidative stress-mediated pulmonary diseases (Liu et al., 2019a). For example, Ye et al. discovered that Lipoxin A4 abates the production of inflammatory factors and reactive oxygen species by enhancing the Nrf2/HO-1 pathway, thus easing acute pancreatitis-induced acute lung injury (Ye et al., 2019). Hence, the modulation of the PI3K/Akt/Nrf2 axis may be a vital target in the treatment of lung injury.

Forkhead transcription factor (FoxO1) is the main transcription factor of fundamental cellular processes, mainly regulating cell proliferation, differentiation, inflammation, and metabolism, etc. (Arcidiacono et al., 2018). On the other hand, NLRP3 forms the NLRP3 inflammasome together with ASC and caspase-1 apoptosis-related dot-like proteins, which mediate various lung injuries (Freeman and Swartz, 2020; Ito et al., 2020; Peng et al., 2020). Interestingly, Tanshinone IIA has been found to reduce blast-induced inflammation, oxidative stress, and cell apoptosis by inhibiting PI3K/Akt/FoxO1, thereby preventing lung embryo cell loss (Liu et al., 2020a). Unfortunately, it is unclear whether the relationship between PI3K/Akt and the FoxO1-NLRP3 axis influences SILI.

In short, KGF-2 has potential effects in ameliorating severe lung injury, and the PI3K/Akt/Nrf2 activation has also been confirmed to protect the lung against inflammation- and oxidative stress-mediated injury. Therefore, we hypothesized that rhKGF-2 alleviates SILI by activating the PI3K/Akt/Nrf2 axis and inhibiting the FoxO1-NLRP3 axis.

## Materials and Methods

### Cell Culture

Human alveolar epithelial cell (HPAEpiC) was purchased from the American Type Culture Collection (ATCC, Rockville, MD, United States) and cultured in the complete medium Dulbecco’s modified Eagle medium/Nutrient Mixture F-12 (DMEM/F-12) (Thermo Fisher Scientific, MA, United States) containing 10% fetal bovine serum (FBS) at 37°C with 5% CO_2_.

Primary rat alveolar epithelial cell was isolated and cultured according to a previous study (Rana et al., 2020). Briefly, the rat lungs were collected in a sterile site. Then the protease solution (300 U/ml collagenase type I, 4 U/ml elastase, 5 U/ml dispase, and 100 μg/ml DNase I in Hanks’ balanced salt solution) was used for the digestion of the lungs at 37°C for 25 min. Next, a continuous digestion with 0.1% trypsin-EDTA and 100 μg/ml deoxyribonuclease I at 37°C for 20 min was performed. The digested lungs were dissociated and made into single-cell suspensions. Then the digestions were passed through a 40 μm mesh size cell strainer. Red blood cells were lyzed, and then macrophages and lymphocytes were removed by incubation with biotinylated anti-CD45 and anti-CD16/32 antibodies (BD Biosciences). The cells were then cultured with DMEM/F-12 medium containing 10% FBS in T75 plates at 37°C overnight to remove fibroblasts. The suspended cells were harvested for further culture and treatment.

### Cell Treatment

The human alveolar epithelial cell (HPAEpiC) cells and primary rat alveolar epithelial cells were incubated with different doses of LPS (5, 10, 20, and 40 μg/ml) [*Escherichia coli* (O111:B4) origin, EC: 297–473–0, Sigma] for 24 h. Then, the cells at the logarithmic growth stage were inoculated into 6-well plates with 5 × 10^5^ cells/well. When the fusion rate of the cells reached 80–90%, different doses of rhKGF-2 (2.5, 5.10 ng/ml) were added for intervention. GSK2126458 (0.5 nM) and AS1842856 (30 nM) (Medchemexpress, New Jersey, United States) was used for treating with HPAEpiC cells for 24 h to inhibit PI3K and FoxO1 respectively.

### 3-(4,5-Dimethylthiazol-2-yl)-2,5-diphenyltetrazolium Bromide Assay

The human alveolar epithelial cell (HPAEpiC) cells and primary rat alveolar epithelial cells were inoculated in 96-well plates at 4 × 10^3^/well and cultured in an incubator at 37°C with 5% CO_2_ for 24 h. They were then treated differently, and the control group was supplemented with an equal volume of PBS. Each group was set with three repetitive wells. After a 24 h culture, 10 μL MTT (5 mg/ml) was added and mixed at low speed for 1 min. Then, the cells were incubated for 4 h at 37°C with 5% CO_2_, and the culture medium was discarded. Afterward, dimethyl sulfoxide (DMSO) was added to lyse the cells. The optical density (OD) value of each well was detected at the wavelength of 570 nm with a microplate reader (Bio-rad, Hercules, CA, United States) after the dissolution of the crystal.

### Flow Cytometry

The human alveolar epithelial cell (HPAEpiC) cells and primary rat alveolar epithelial cells were trypsinized by EDTA-free trypsin (Beyotime, Shanghai, China) and centrifuged at 2,000 rpm (340 g) for 5 min, and the medium was discarded. After washing with PBS, the cells were collected for cryopreservation. Then, they were treated with an AnnexinV-PE/7-AAD apoptosis kit (Southern Biotechnology, Birmingham, Al, United States). FCM (Bechman Coulter, CA) was implemented on cells labeled with AnnexinV-PE and 7-AAD to test cell apoptosis. The experiment was repeated three times.

### Enzyme-Linked Immunosorbent Assay

After treatment with various factors, the rat lung homogenate was made, which was then centrifuged (400 g, 20 min) to retain the supernatant. Meanwhile, the human alveolar epithelial cell (HPAEpiC) cells and primary rat alveolar epithelial cells were centrifuged (340 g, 10 min), and the cell supernatant was collected. The levels of tumor necrosis factor (TNF-α), interleukin-1β (IL-1β), interleukin-6 (IL-6), high sensitivity C-reactive protein (hsCRP), malondialdehyde (MDA), superoxide dismutase (SOD) and glutathione peroxidase (GSH-PX) in rat lungs or alveolar epithelial cells were determined referring to the manufacturer’s instructions (Nanjing Biotechnology Co., Ltd., Nanjing, China).

### Western Blot

The rat lung tissues were collected for homogenization, lyzed and centrifugated (14,000 rpm for 30 min at 4°C) to obtain total protein. The total protein of human alveolar epithelial cell (HPAEpiC) cells and primary rat alveolar epithelial cells was extracted with the RIPA lysate (Beyotime, Shanghai, China). The protein concentration was examined by the BAC Protein Determination Kit (Beyotime, Shanghai, China). The proteins were separated by 10% polyacrylamide gel electrophoresis and transferred to polyvinylidene fluoride (PVDF) membranes (Millipore, Bedford, MA, United States). Afterward, the membranes were blocked with 5% skim milk and incubated with the primary antibodies of p-PI3K (1:1,000 dilutions, CST, United States), PI3K (1:1,000 dilutions, CST, United States), Anti-AKT1 (phospho S473) antibody (1:1,000, ab38449, Abcam, United States), Anti-AKT antibody (1:1,000, ab8805, Abcam, United States), Anti-Nrf2 antibody (1:1,000, ab137550, Abcam, United States), Anti-β-actin antibody (1:1,000, ab8224, Abcam, United States), Anti-FoxO1 antibody (1:1,000, ab39607, Abcam, United States), Anti-FoxO1 (phospho S256) antibody (1:1,000, ab131339, Abcam, United States), Anti-NLRP3 antibody (1:1,000, ab263899, Abcam, United States), Anti-ASC antibody (1:1,000, ab151700, Abcam, United States), Anti-Caspase1 antibody (1:1,000, ab207802, Abcam, United States), Anti-HO-1 antibody (1:1,000, ab68477, Abcam, United States), Anti-NQO-1 antibody (1:1,000, ab80588, Abcam, United States), Anti- Histone H3 antibody (1:1,000, ab1791, Abcam, United States) at 4°C overnight. After the primary antibodies were eluted on the next day, the membranes were incubated with the horseradish peroxidase-labeled Goat Anti-Rabbit IgG H&L (1:3,000, ab97051, Abcam, United States) at room temperature for 1 h. Then, the membranes were cleared three times. Quantity One Software (BioRad, Hercules, CA, United States) was used to detect staining intensity.

### Intracellular Reactive Oxygen Species Level Detection

Intracellular ROS level was detected by the DCFDA/H2DCFDA-Cellular ROS Assay kit (ab113851, Abcam, United States). Briefly, the human alveolar epithelial cell (HPAEpiC) cells and primary rat alveolar epithelial cells were seeded in 24-well plates (5 × 10^5^ cells/well). After being incubated at 37°C and treated with LPS and/or rhKGF2 for 24 h, the cells were washed with the 1× buffer and then incubated with the diluted DCFDA Solution at 37°C for 45 min. Next, the cells were washed with the 1× buffer for 2 times. Finally, the ROS expression were observed and measured under a fluorescence microscope (Olympus, Japan).

### Animal Model

A total of sixty adult Sprague-Dawley rats (30 male and 30 female, body wight 150–250 g) aged 7–8 weeks were bought from the Animal Experiment Center of NanChang University (Nanchang, China) and kept in a specific pathogen-free (SPF) environment. Smoke inhalation induced lung injury (SILI) model was constructed referring to a previous study with few changes (Yilin et al., 2015). Briefly, the smoke was produced from heated mixture of 150 g of dry wood shavings and 30 ml of kerosene. The smoke was wafted from the pot to a 20 × 20 × 20 cm holding cage using an air blower. This cage was kept at a temperature of 35 ± 2°C and was equipped with transparent glass on the top with a light/(bulb) in each of the four corners. The transparent glass provided visibility for the determination of the optimum smoke density. After the cage was filled with smoke, 40 rats were placed in the holder for 10 min, and subsequently taken out of the holder for 5 min. This cycle was repeated three times. The O_2_ and CO_2_ concentration inside the chamber was determined using a gas chromatography (TRACE™ 1300, Thermo Scientific™). The arrange O2 concentration was 2.36 ± 0.29%, and the CO_2_ concentration was 17.26 ± 1.21%. The above steps were repeated three times, each time every day within 3 days. The rats were randomized into 6 groups (including sham, rhKGF-2, SILI, SILI + rhKGF-2, SILI + GSK2126458, SILI + AS1842856, SILI + rhKGF-2+GSK2126458, SILI + rhKGF-2+AS1842856). Rats in the Sham group did not inhale smoke and received solvent administration. rhKGF-2 was inhaled by the rats (2 mg/kg dissolved in 5 ml PBS) with a pediatric atomizer, 9 o’clock in the morning every day (for 20 min). GSK2126458 (300 μg/kg, Medchemexpress, New Jersey, United States) and AS1842856 (10 μg/kg, Medchemexpress, New Jersey, United States) were dealt with the rats by intraperitoneal injection (once every 2 days for 3 times) (Cui et al., 2018; Liu et al., 2020a). After treatment for consecutive 7 days, the rats were intraperitoneally injected with pentobarbital sodium (50 mg/kg) for anesthesia. After anesthesia, aortic blood was extracted. Then, the rats were sacrificed, and their tracheal carinas and lungs were taken. The experimental procedure followed the Guidelines for the Care and Use of Laboratory Animals published by the National Institutes of Health (NIH) (Publication No. 85-23, 2011). The experimental conductors tried their best to avoid unnecessary suffering on the rats when constructing the model. The animal study has been approved by ethics committee of the First Affiliated Hospital of Nanchang University.

### Blood-Gas Analysis

2 ml of rat blood was collected from the abdominal aorta of the rats. The partial pressure of oxygen (PaO_2_), the partial pressure of carbon dioxide (PaCO_2_), and the oxygenation index (PaO_2_/FiO_2_ ratio) were measured with a blood gas analyzer (Instrumentation Laboratory, Lexington, MA).

### Hematoxylin-Eosin (HE) Staining

The rat lung tissue was fixed with 4% formalin, cleaned, dehydrated, paraffin-embedded, and sectioned (5 μm-thickness). Then, the sections were dyed with H&E, dehydrated with gradient ethanol, transparentized with xylene, and mounted with resin. The morphological damage and changes of lung tissues were observed by an optical microscope (Olympus, Tokyo, Japan). The degree of inflammatory cell infiltration, hemorrhage, interstitial and alveolar edema, and the thickness of alveolar septum were included as injury evaluation. The scoring criteria (Qi et al., 2014) of lung injury are as follows: 0: no injury; 1: the area of injury is more than 25%; 2: the area of injury is more than 50%; 3: the area of injury is more than 75%; 4: diffuse lung injury. The total score is the sum of five standard scores.

### Determination of Pulmonary Edema

After the lung was removed from the thorax, it was thoroughly cleaned with normal saline. Then, the lung surface was sucked dry with clean gauze, and the lung was weighed (wet weight/W) with a precision balance. Subsequently, the lung sample was heated in a constant temperature oven at 80°C for 48 h and weighed (dry weight/D). The pulmonary edema index = W/D.

### Terminal-Deoxynucleoitidyl Transferase Mediated Nick End Labeling (TUNEL) Assay

The rat lung tissues were collected, fixed with 4% paraformaldehyde, embedded in paraffin and sectioned (4 μm). Then the TUNEL assay was performed according to the instructions of TUNEL In Situ Cell Death Detection Kit (cat.no.12156792910, Roche, Basel, Switzerland) and referred to (Liu et al., 2017). The sections were deparaffinized, and then rehydrated with decreasing concentrations of ethanol. After that, the sections were covered with proteinase K solution for 15–20 min (at room temperature). Once the sections were washed with PBS (for 3 times, 5 min each time), the sections were incubated with the TUNEL reaction mixture and incubated for 1 h in the dark. DAB substrate was used to react with the sections (at room temperature for 10 min). Finally, the images were observed under a microscope (Olympus, Tokyo, Japan) to analyze cell apoptosis (200x). Then we calculated the number of TUNEL-positive cells: positive cell rate = (number of positive cells/number of all cells) × 100%.

### Data Process

The measurement data were presented as mean ± standard deviation (x ± s). GraphPad Prism 8 Software (GraphPad Software, Inc., San Diego, CA, United States) was used for statistical processing. Student’s *t* test was employed to analyze the statistical significance between two groups. The one-way analysis of variance was applied to compare the data among multiple groups with Tukey post hoc test. *p* < 0.05 represented statistical significance.

## Results

### rhKGF-2 Prevented LPS-Induced Lung Endothelial Cell Injury *via* Repressing Inflammation and Oxidative Stress

**FIGURE 1:**
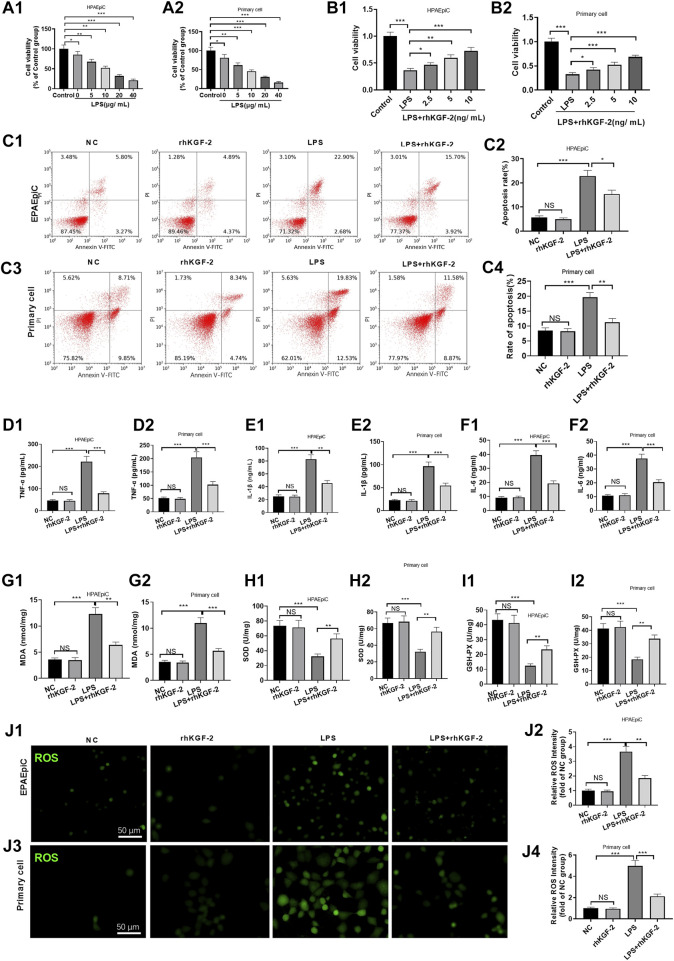
The effect of rhKGF-2 on the cell viability, apoptosis, inflammation, oxidative and ROS generation of alveolar epithelial cells with LPS treatment. The HPAEpiC cells and primary rat alveolar epithelial cells were treated with LPS (5–20 μg/ml) and rhKGF-2 (2.5–10 ng/ml) for 24 h. **A,B**: Cell viability was determined by the MTT assay. The HPAEpiC cells and primary rat alveolar epithelial cells were treated with LPS (10 μg/ml) and rhKGF-2 (10 ng/ml) for 24 h. **A1, A2, B1, B2**: MTT assay were implemented to verify cell viability. The cell viability was calculated as the relative fold change of control group; **C1–C4**: FCM was conducted to analyze apoptosis. The rate of cell in the right upper quadrant was counted. **D–O**: The expression of TNF-α, IL-1β, IL-6, MDA, SOD, and GSH-PX in HPAEpiC cells and primary rat alveolar epithelial cells were examined by ELISA. The *Y*-axis shows the content of the detected mediators. *p*: The cellular ROS (marked by green color) level in HPAEpiC cells and primary rat alveolar epithelial cells was detected using the DCFDA/H2DCFDA-Cellular ROS Assay kit. The *X*-axis indicates the relative fluorescence intensity of ROS of NC group. Data are expressed as mean ± SD. All experiments were repeated for three times. Data difference was analyzed via ANOVA, and Student’s t test. NS*P*>0.05, *p<0.05, **p<0.01, ***p<0.001. N = 3.

LPS was used for inducing injury on human alveolar epithelial cell (HPAEpiC) cells and primary rat alveolar epithelial cells (primary cell). HPAEpiC cell and primary cell were treated with different doses of LPS (5–40 μg/ml) and rhKGF-2 (2.5–10 ng/ml). We found that LPS markedly decreased cell viability (compared with NC group, Figures 1A1,A2). However, rhKGF-2 dose-dependently enhanced the viability of HPAEpiC cell and primary cell (compared with LPS group, Figures 1B1,B2). Thus, the appropriate doses (20 μg/ml LPS, 10 ng/ml rhKGF-2) were chosen for the latter experiment. FCM were further adopted to analyze cell apoptosis. It turned out that LPS enhanced cell apoptosis, while rhKGF-2 treatment significantly reduced apoptosis-mediated by LPS (vs. LPS group, *p* < 0.05, Figures 1C1–C4). ELISA was employed to analyze the inflammation and oxidative stress in HPAEpiC cell and primary cell. As a result, LPS induced the increased release of TNF-α, IL-1β, IL-6, MDA, and attenuated the levels of SOD and GSH-PX in both HPAEpiC cell and primary cell, while the LPS + rhKGF-2 group had the reverse results and eased LPS-induced damage and inflammation (compared with LPS group, *p* < 0.05, Figures 1D–I). What’s more, the cellular ROS level in HPAEpiC cell and primary cell was detected. The results turned out that LPS significantly promoted ROS level in the cells. With the treatment of rhKGF-2, the ROS level was significantly attenuated (compared with LPS group, *p* < 0.05, Figures 1J1,J2). These results indicated that rhKGF-2 had the potential to protect alveolar epithelial cells, maintain cell survival, and reduce inflammation and oxidative stress.

### rhKGF-2 Regulates PI3K/Akt/Nrf2 and FoxO1-NLRP3 Expression *in vitro*

**FIGURE 2:**
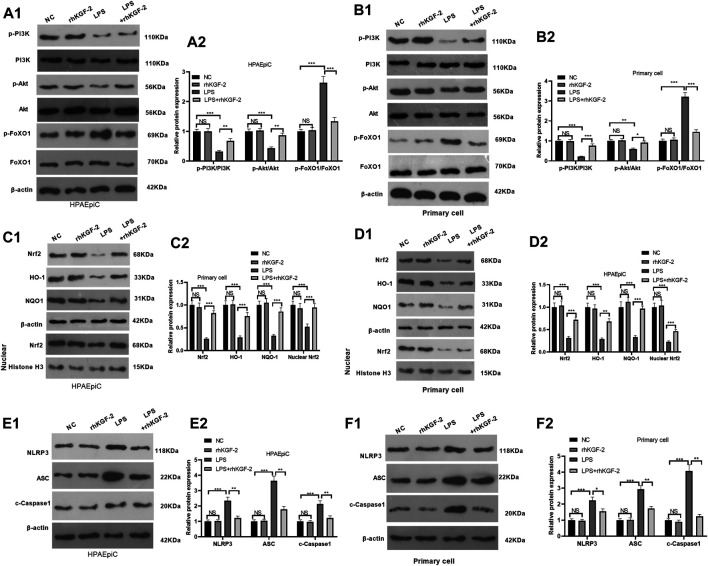
The effect of rhKGF-2 on the expression of PI3K/Akt, Nrf2/HO-1/NQO1, and FoxO1-NLRP3-ASC-Caspase1 inflammasome. The HPAEpiC cells and primary rat alveolar epithelial cells were treated with LPS (10 μg/ml) and/or rhKGF-2 (10 ng/ml) for 24 h. **A1-A2**: WB was carried out to determine the expression of PI3K/Akt and FoxO1 in HPAEpiC cells. **B1-B2**: WB was carried out to determine the expression of PI3K/Akt and FoxO1 in primary rat alveolar epithelial cells. **C1-C2**. The protein levels of Nrf2, HO-1 and NQO1 in the HPAEpiC cells were detected by WB. **D1-D2**. The protein levels of Nrf2, HO-1 and NQO1 in the primary rat alveolar epithelial cells were detected by WB. **E1-E2**. WB was conducted to detect NLRP3-ASC-Caspase1 in HPAEpiC cells. **F1-F2**. WB was conducted to detect NLRP3-ASC-Caspase1 in primary rat alveolar epithelial cells. The p-PI3K, p-AKT and p-FoxO1 were calculated using PI3K, AKT and FoxO1 as internal control, respectively. β-actin was the internal control of Nrf2, HO-1, NQO1 and NLRP3-ASC-Caspase1. Nuclear Nrf2 was calculated using Histone H3 as internal control. The relative protein expression (*Y*-axis) was calculated as fold change of NC group. The *X*-axis showed the grouping names. Data are expressed as mean ± SD. All experiments were repeated for three times. Data difference was analyzed via ANOVA, and Student's t test. NS*P*>0.05, **p < 0.01, ***p < 0.001. N = 3.

In order to reveal the downstream mechanism of rhKGC-2 on alveolar epithelial cells, the expressions of PI3K/Akt pathway, Nrf2/HO-1/NQO1 and FoxO1-NLRP3-ASC-Caspase1 inflammasome in HPAEpiC cell and primary cell were detected by WB. The results showed that LPS reduced the phosphorylation of PI3K/Akt, inhibited cellular and nuclear Nrf2 level and attenuated HO-1/NQO1 expression in HPAEpiC cell and primary cell (*p* < 0.05, Figures 2A1–D2). However, the rhKGF-2 treatment activated PI3K/Akt pathway, enhanced Nrf2 nuclear translocation, and promoted HO-1/NQO1 expression (*p* < 0.05 compared with LPS group, Figures 2A1–D2). Moreover, we evaluated FoxO1 and NLRP3-ASC-Caspase1 inflammasome in HPAEpiC cell and primary cells. It was found that LPS induced enhanced phosphorylation of FoxO1 and NLRP3-ASC-Caspase1 inflammasome activation (compared with con group, *p* < 0.05, Figures 2A1-B2, E1-F2). The treatment of rhKGF-2 significantly mitigated phosphorylation of FoxO1 and NLRP3-ASC-Caspase1 inflammasome activation (compared with LPS group, *p* < 0.05, Figures 2A1-B2, E1-F2). These findings confirmed that rhKGF-2 reversed LPS-mediated effects via activating PI3K/Akt/Nrf2 and downregulating FoxO1-NLRP3-ASC-Caspase1 inflammasome *in vitro*.

### Downregulating the PI3K/Akt Pathway and FoxO1 Pathway Partly Altered rhKGF-2 Mediated Effects

**FIGURE 3:**
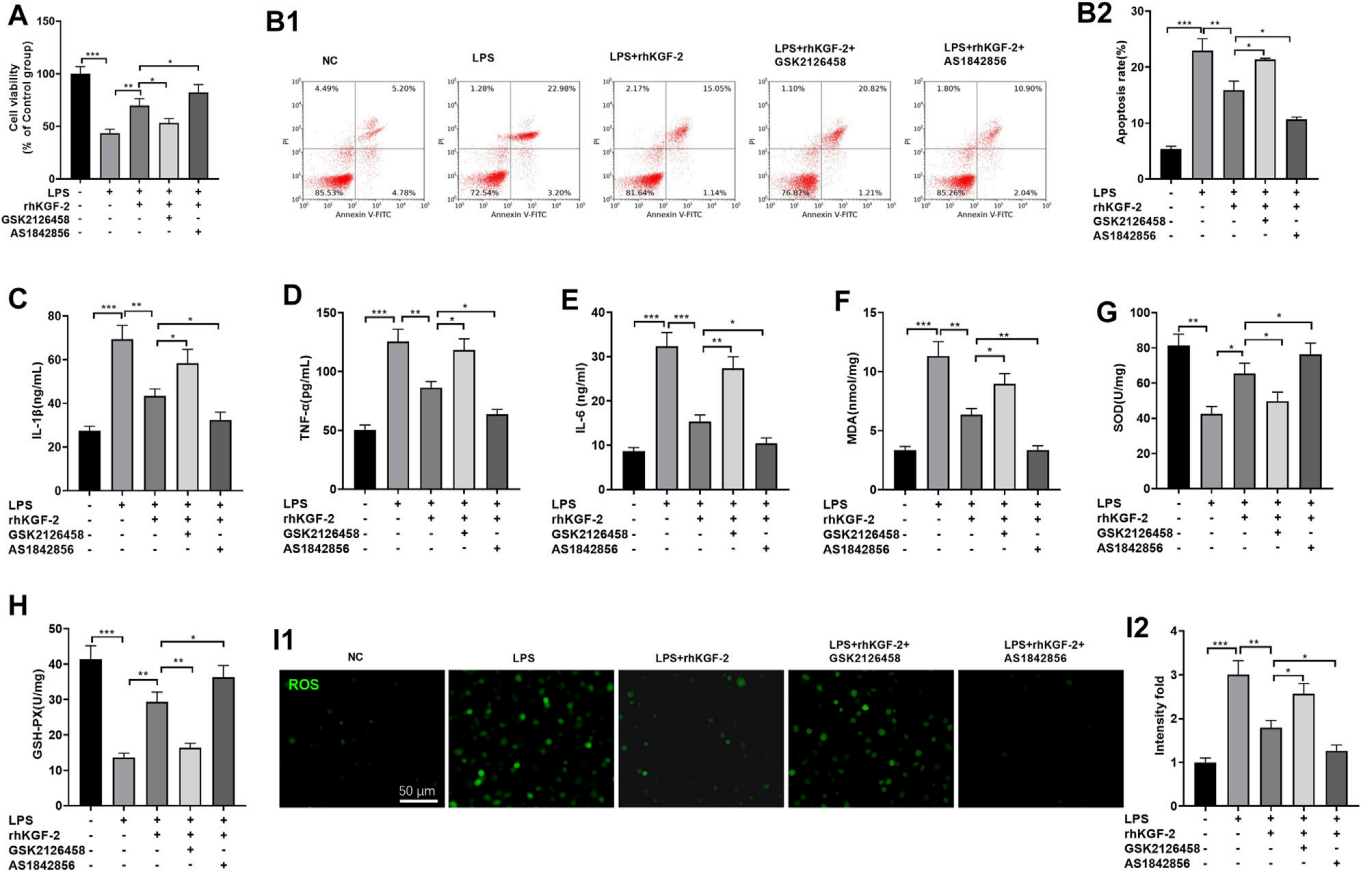
The effect of PI3K inhibitor and FoxO1 inhibitor on rhKGF-2-mediated effect on HPAEpiC cells. LPS-treated HPAEpiC cells were adopted to construct a lung cell injury model, and then rhKGF-2 (10 ng/ml), GSK2126458 (0.5 nM) and AS1842856 (30 nM) were added for treatment for 24 h. **(A)**: MTT assay was implemented to verify HPAEpiC viability. The cell viability was calculated as the relative fold change of control group. **B1-B2**. Flow cytometry was performed to verify HPAEpiC apoptosis. The rate of cell in the right upper quadrant was counted as apoptotic cells. **C–H**: ELISA was conducted to detect the expression of TNF-α **(C)**, IL-1β **(D)**, IL-6 **(E)**, MDA **(F)**, SOD **(G)**, and GSH-PX. **(H)**. The *Y*-axis shows the content of the detected mediators. **(I)**. The cellular ROS level was detected using the DCFDA/H2DCFDA-Cellular ROS Assay kit. The *X*-axis indicates the relative fluorescence intensity of ROS of NC group. Data are expressed as mean ± SD. All experiments were repeated for three times. Data difference was analyzed via ANOVA, and Student's *t* test. *p<0.05, **p<0.01, ***p<0.001. N = 3.

HPAEpiC cells were treated with LPS, GSK2126458 (PI3K inhibitor) or AS1842856 (FoxO1 inhibitor). Then the HPAEpiC cell viability, apoptosis, inflammatory and oxidative stress respones were evaluated. The results of MTT assay and FCM showed that compared with the LPS + rhKGF-2G group, SK2126458 administration dampened the cytoprotective effects of rhKGF-2 against LPS, resulting in decreased cell viability and increased apoptosis. Moreover, the AS1842856 administration significantly facilitated the effects of rhKGF-2, manifested by increasing cell viability and inhibiting apoptosis (Figures 3A,B2). The levels of inflammatory factors and oxidative stress markers were compared by ELISA. Interestingly, compared with the LPS + rhKGF-2 group, TNF-α, IL-1β, IL-6, MDA and ROS level were up-regulated, while SOD and GSH-PX were down-regulated in the LPS + rhKGF-2+GSK2126458 group. On the contrary, TNF-α, IL-1β, IL-6, MDA and ROS level were down-regulated, while SOD and GSH-PX were enhanced in the LPS + rhKGF-2+AS1842856 group (*p* < 0.05, Figures 3C–I2). Thus, rhKGF-2 protected lung cells from inflammation and oxidative stress *in vitro*, and stabilizing cell survival dependently through PI3K/Akt/FoxO1 pathway and NLRP3 inflammasome.

### Repressing PI3K Attenuated rhKGF-2 Induced PI3K/Akt/Nrf2 Pathway Activation and FoxO1-NLRP3 Downregulation

**FIGURE 4:**
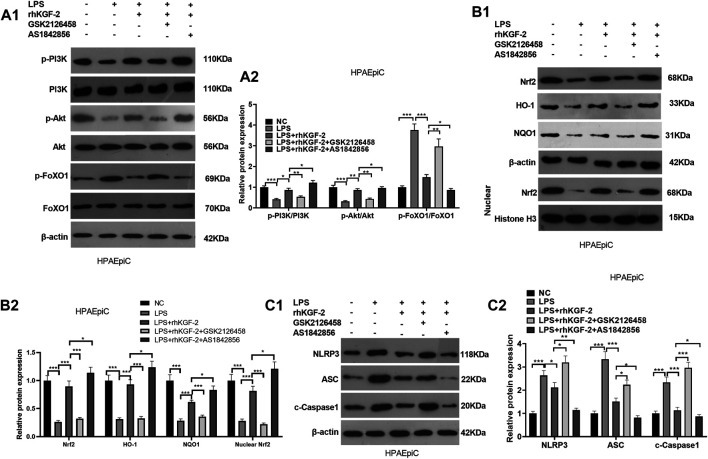
The effect of PI3K inhibitor and FoxO1 inhibitor on the expression of PI3K/Akt, Nrf2/HO-1/NQO1, and FoxO1-NLRP3-ASC-Caspase1 inflammasome. BEAS-2B cells were treated with LPS (10 μg/ml), rhKGF-2 (10 ng/ml), GSK2126458 (0.5 nM) and/or AS1842856 (30 nM) for 24 h. **A1-A2**: WB was carried out to determine the expression of PI3K/Akt and FoxO1 in HPAEpiC cells. **B1-B2**. The protein levels of Nrf2, HO-1 and NQO1 in the HPAEpiC cells or nuclear were detected by WB. Nuclear Nrf2 was calculated using Histone H3 as internal control. **C1-C2**. WB was conducted to detect NLRP3-ASC-Caspase1 in HPAEpiC cells. The relative protein expression (*Y*-axis) was calculated as fold change of NC group. Data are expressed as mean ± SD. All experiments were repeated for three times. Data difference was analyzed via ANOVA, and Student's t test. *p<0.05, **p<0.01, ***p<0.001. N = 3.

HPAEpiC cells were treated with LPS, rhKGF-2, GSK2126458 (PI3K inhibitor) and/or AS1842856 (FoxO1 inhibitor). Then we detected PI3K/Akt/Nrf2 pathway and FoxO1-NLRP3 axis *via* WB. It was found that GSK2126458 treatment attenuated PI3K/Akt phosphorylation, restrained nuclear translocation of Nrf2 and cellular HO-1 and NQO1, but promoted FoxO1-NLRP3-ASC-Caspase1 inflammasome expression (compared with the LPS + rhKGF-2 group, Figures 4A1–C2). By contrast, PI3K/Akt phosphorylation and Nrf2/HO-1/NQO1 levels were increased and FoxO1-NLRP3-ASC-Caspase1 inflammasome was inhibited with AS1842856 treatment (compared with the LPS + rhKGF-2 group, Figures 4A1–C2). Thus, rhKGF-2 exerted its role in relieving HPAEpiC cell injury *via* regulating the PI3K/Akt/FoxO1 pathway and NLRP3 inflammasome.

### rhKGF-2 Improved Lung Injury in the Rat SILI Model

**FIGURE 5:**
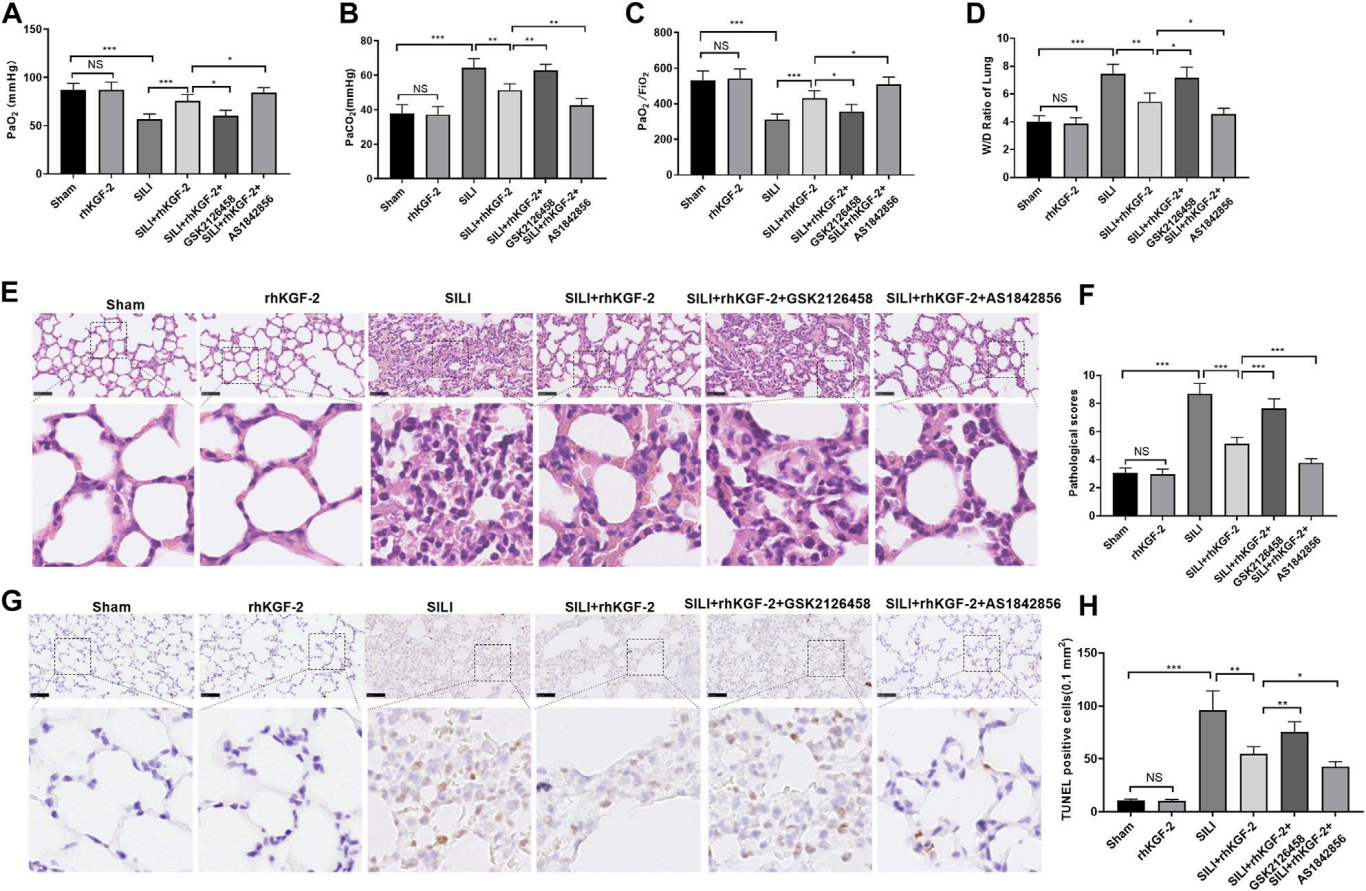
The role of rhKGF-2 SILI rat model. The SILI rat model was established by inhalation of smoke. Then, rhKGF-2 (10 ng/kg body weight), GSK2126458 (300 μg/kg body weight) and AS1842856 (10 μg/kg body weight) were used for treating the SILI rats, the same volume of saline was administered on the rat in the sham group. **(A–C)**. The arterial blood of rats was extracted to detect the values of PaO_2_, PaCO_2_, PaO_2_/FiO_2_ to analyze the pulmonary function. The *Y*-axis of panel A and B represents the partial pressure of O_2_ and CO_2_ (mmHg). The *Y*-axis of panel C represents the Oxygenation index (the ratio of arterial partial pressure of oxygen to inhaled oxygen concentration) (mmHg). **(D)**. The wet weight (W) and dry weight of the rat lung were measured, and the W/D ratio was calculated to evaluate the pulmonary edema. **(E)**. HE staining was performed to observe the pathological changes of rat lung in each group. **(F)**. The pathological scores of rat lung in each group were counted. **(G)**. TUNEL assay was implemented to analyze apoptosis in the lung tissues. **(H)**. The number of TUNEL-positive cells in an area of 0.1 mm^2^ was calculated. Scale bar = 50 μm. Data are expressed as mean ± SD. All experiments were repeated for three times. Data difference was analyzed via ANOVA, and Student's t test. NS*P*>0.05, *p<0.05, **p<0.01, ***p<0.001. Five rats were involved in each group (N = 5).

A SILI rat model was constructed and dealt with rhKGF-2. First, we examined the pathophysiological changes in the rat lung tissue. Blood gas analysis showed that the blood gas of the sham group and rhKGF-2 group remains at a normal level, while the SILI group had declined PaO_2_ and PaO_2_/FiO_2_ and elevated PaCO_2_ level. However, rhKGF-2 significantly inhibited the increase of PaCO_2_ and the decrease of PaO_2_ and PaO2/FiO_2_ induced by smoke inhalation (*p* < 0.05 compared with SILI group, Figures 5A–C). In comparison with the SILI + rhKGF-2 group, the addition of GSK2126458 increased PaCO_2_ but decreased the level of PaO_2_ and PaO2/FiO_2_ in the blood. Oppositely, AS1842856 administration on the basis of the rhKGF-2 treatment synergistically enhanced the effect of rhKGF-2 (*p* < 0.05, Figures 5A–C). The lung edema was detected by W/D method and the histopathological changes were analyzed by HE staining, and TUNEL assay. As a result, the lung tissues of the sham group, rhKGF-2 group had normal appearance without obvious edema and apoptosis. In contrast, the SILI group had severe pulmonary edema, thickened alveolar walls, swollen vascular endothelial cells, a large number of infiltrating inflammatory cells, oozing red blood cells, and increased apoptosis (*p* < 0.05, Figures 5D–H). On the contrary, the rhKGF-2 treatment significantly inhibited the smoke inhalation-induced pulmonary edema and improved the pathological changes and apoptosis of lung tissues. Meanwhile, GSK2126458 partially offset the protective effect of rhKGF-2, while AS1842856 strengthened the effect of rhKGF-2 (*p* < 0.05, Figures 5D–H). These results suggested that rhKGF-2 alleviated SILI, while the PI3K and FoxO1 inhibitor antagonized and promoted the effects of rhKGF-2, respectively.

### rhKGF-2 Alleviated the Pro-inflammatory and Oxidative Stress Responses in the Rat SILI Model

**FIGURE 6:**
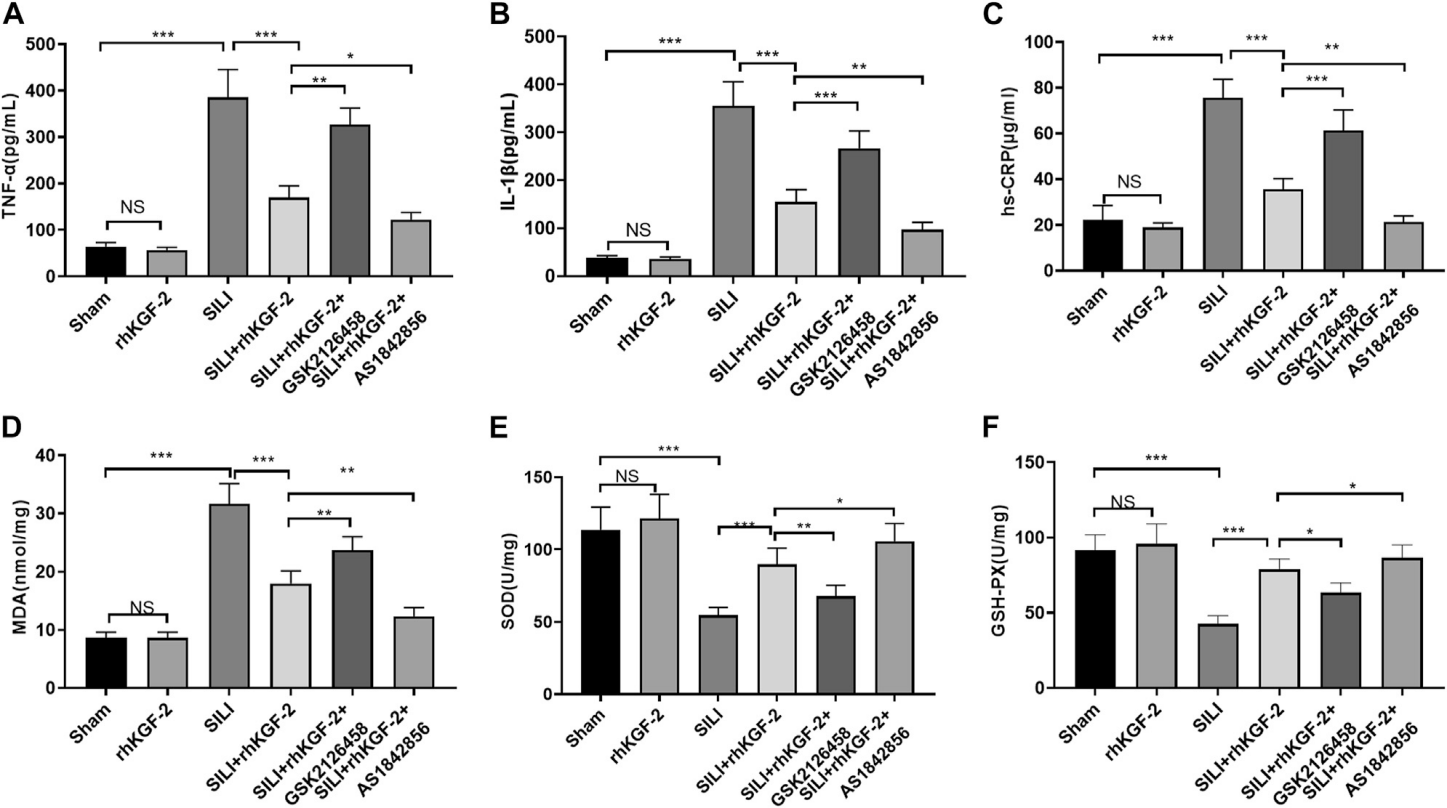
rhKGF-2 eased the inflammation and oxidative stress in the SILI rat model. The SILI rat model was established by inhalation of smoke. Then, rhKGF-2 (10 ng/kg body weight), GSK2126458 (300 μg/kg body weight) and AS1842856 (10 μg/kg body weight) were used for were used for treating the SILI rats, the same volume of saline was administered on the rat in the sham group. A–F: The release of pro-inflammatory cytokines TNF-α **(A)**, IL-1β **(B)**, hs-CRP **(C)**, and the levels of oxidative stress markers MDA **(D)**, SOD **(E)**, and GSH-PX. **(F)** in the lung of rats were determined by ELISA. The *Y*-axis shows the content of the detected mediators. The *X*-axis shows the grouping name. Data are expressed as mean ± SD. All experiments were repeated for three times. Data difference was analyzed *via* ANOVA, and Student’s t test. NS*P*>0.05, *p<0.05, **p<0.01, ***p<0.001. Five rats were involved in each group (N = 5).

The levels of pro-inflammatory cytokines and oxidative stress markers in rats were tested by ELISA. Interestingly, the pro-inflammatory cytokines or markers, including TNF-α, IL-1β, hs-CRP, and the pro-oxidative stress MDA were increased, while the antioxidant mediators SOD and GSH were inhibited in the SILI group (*p* < 0.05 vs. Sham group, Figures 6A–F). In addition, the rhKGF-2 treatment reduced the SILI-induced up-regulation of TNF-α, IL-1β, hs-CRP, and MDA, and the down-regulation of SOD and GSH (*p* < 0.05 vs. SILI group, Figures 6A–F). The administration of GSK2126458 partially offset the effects of rhKGF-2, while AS1842856 facilitated the protective effects of rhKGF-2 (*p* < 0.05 vs. SILI + rhKGF-2 group, Figures 6A–F). The above results confirmed that rhKGF-2 resisted inflammation and oxidative stress in SILI, and GSK2126458 and AS1842856 respectively inhibited and increased the protective effects.

### rhKGF-2 Upregulated PI3K/Akt Pathway and Inhibited FoxO1-NLRP3 Inflammasome

**FIGURE 7:**
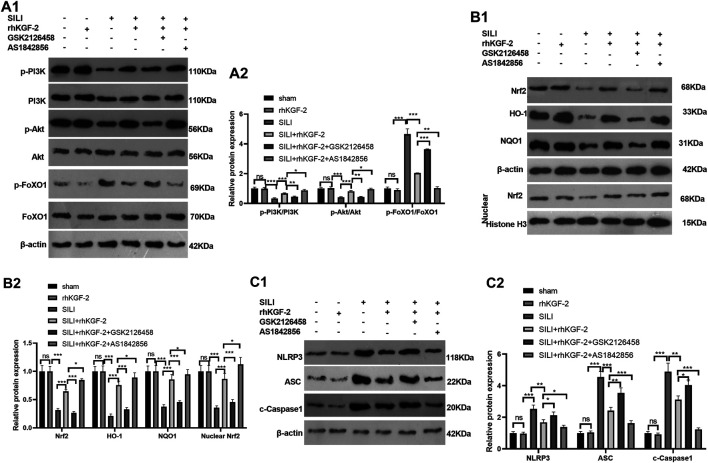
rhKGF-2 has a role in regulating the activation of PI3K/Akt pathway, Nrf2/HO-1/NQO1 pathway and FoxO1-NLRP3-ASC-Caspase1 inflammasome *in vivo.* The SILI rat model was established by inhalation of smoke. Then, rhKGF-2 (10 ng/kg body weight), GSK2126458 (300 μg/kg body weight) and AS1842856 (10 μg/kg body weight) were used for were used for treating the SILI rats, the same volume of saline was administered on the rat in the sham group. Left lung tissues of rats in each group were collected for detection. **A1-A2**: WB was carried out to determine the expression of PI3K/Akt and FoxO1 in the lung tissues. **B1-B2**. The protein levels of Nrf2, HO-1 and NQO1 in the lung tissues. or nuclear were detected by WB. Nuclear Nrf2 was calculated using Histone H3 as internal control. **C1-C2**. WB was conducted to detect NLRP3-ASC-Caspase1 in the lung tissues. The relative protein expression (*Y*-axis) was calculated as fold change of sham group. Data are expressed as mean ± SD. All experiments were repeated for three times. Data difference was analyzed via ANOVA, and Student's t test. NS*P*>0.05, *p<0.05, **p<0.01, ***p<0.001. Five rats were involved in each group (N = 5).

**FIGURE 8:**
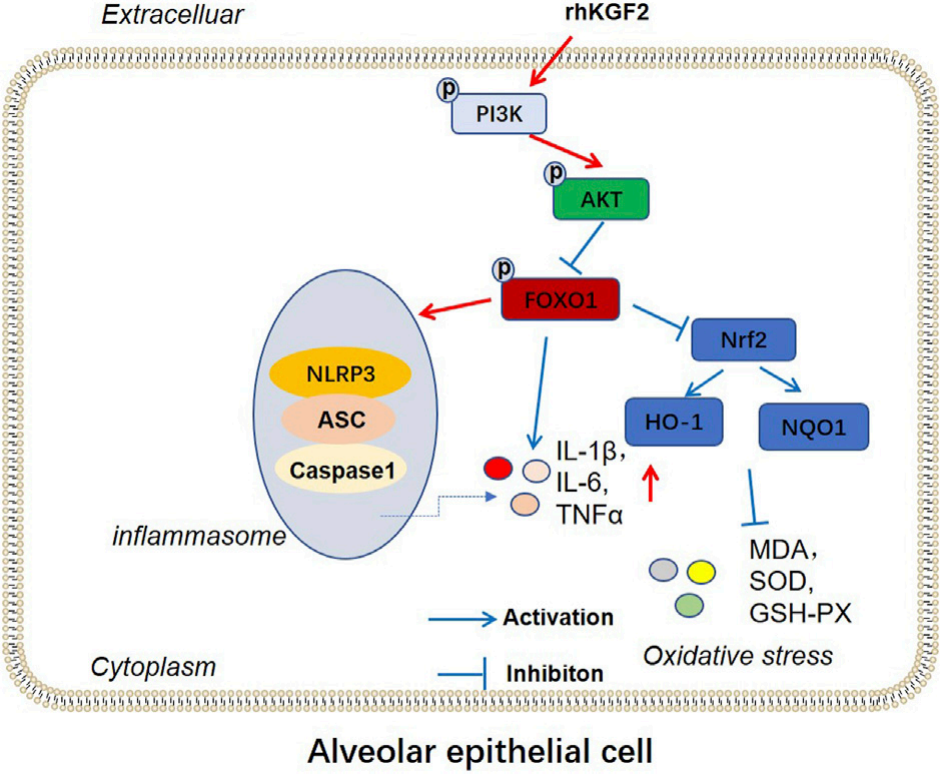
The mechanism diagram. rhKGF2 enhanced the activation of PI3K/AKT pathway, inhibited the phosphorylation of FoxO1, thus repressing NLRP3-ASC-Caspase1 inflammasome and promoted Nrf2/HO-1/NQO1 axis. Finally, rhKGF2 repressed inflammation and oxidative stress of alveolar epithelial cells.

The profiles of PI3K/Akt/FoxO1 pathway, Nrf2/HO-1/NQO1 and NLRP3-ASC-Caspase1 inflammasome was verified by WB to clarify the potential downstream signaling pathway by which rhKGF-2 prevented SILI. The results revealed that p-PI3K, p-Akt, nuclear Nrf2, HO-1 and NQO1 were down-regulated following the insult of SILI, while p-FoxO1 and NLRP3-ASC-Caspase1 inflammasome were up-regulated in the SILI group compared with that in the sham group (*p* < 0.05, Figure 7A1–C2). In addition, rhKGF-2 enhanced p-PI3K, p-Akt, nuclear Nrf2, HO-1 and NQO1, and down-regulated p-Foxo1 and NLRP3-ASC-Caspase1 inflammasome (vs.SILI group, *p* < 0.05, Figures 7A–D). With the addition of PI3K inhibitor GSK2126458, p-PI3K, p-Akt, nuclear Nrf2, HO-1 and NQO1 levels were down-regulated, while p-FoxO1 and NLRP3-ASC-Caspase1 inflammasome were up-regulated compared with those in the SILI + rhKGF2 group (Figures 7A1–C2). However, the treatment of FoxO1 inhibitor AS1842856 enhanced the activation of PI3K/AKT, upregulated nuclear Nrf2, HO-1 and NQO1 and repressed p-FoxO1, NLRP3-ASC-Caspase1 inflammasome (vs.SILI + rhKGF2 group, *p* < 0.05, Figures 7A1–C2). These findings illustrated that rhKGF-2 induced the activation of PI3K/Akt pathway but inactivated FoxO1-NLRP3 axis (Figure 8).

## Discussion

SILI and severe skin burn are the key factors for fire-related deaths, which can be as high as 86% if both the factors occur (Peters, 1981). What’s more, those patients often develop into progressive respiratory failure associated with pulmonary oedema and even acute respiratory distress syndrome (ARDS) is a pivotal determinant of mortality (Traber et al., 2007). According to a follow-up investigation, the chest computed tomography (CT) examination of firefighters with high-intensity World Trade Center (WTC) exposure showed that they were with bronchial wall thickening, emphysema and respiratory symptoms (Liu et al., 2019b). Studies have demonstrated that the stimulation of the respiratory system by a range of harmful chemicals such as carbon monoxide, cyanide and malondialdehyde, can lead to dyspnea and even suffocation, which is linked to oxidative stress/antioxidant stress imbalance and inflammatory cascades (David et al., 2009). In this paper, it was found that rhKGF-2 exerts its anti-inflammatory and antioxidant effects, thus reducing SILI through both *in-vitro* and *in-vivo* experiments.

Inflammation is a complex biological response of the body to harmful stimuli, which aims to resist cell damage and initiate repair. At the same time, inflammation causes oxidative damage to alveolar epithelial cells through the formation of reactive oxygen species and reactive nitrogen, and a persistent imbalance of oxidative stress causes cell death (Valavanidis et al., 2013; Yonezawa et al., 2016; Shao et al., 2019; Zhong et al., 2020). KGF-2, also known as FGF10, is very important for tissue development and a stable environment. It was first isolated from rat embryos by Yamasaki et al. (1996). Multiple researches have shown that KGF-2 prevents serious lung diseases. For instance, FGF-10 reduces the particulate matter-induced airway inflammation by inhibiting the release of inflammatory factors and cell apoptosis (Liu et al., 2019c). Also, FGF-10 plays an anti-inflammatory and cell-protective role by inhibiting the HMGB1-TLR4 pathway, avoiding contact particulate matter-induced lung injury (Liu et al., 2020b). In addition, Lin et al. found that intravenous drip of FGF-10 in the rat lung mobilizes lung mesenchymal stem cells, thus reducing the recruitment of inflammatory cytokines and preventing acute lung injury caused by trauma or infection (Tong et al., 2016). Besides, intratracheal infusion of KGF-2 significantly improves pulmonary edema and inflammation and alleviates ventilator-induced acute lung injury (Bi et al., 2014). Here, we constructed the SILI rat model and found that after inhalation of a large amount of smoke, the rats’ respiratory function decreased sharply, accompanied by serious pathological changes, such as pulmonary edema, alveolar wall thickening, vascular endothelial cell swelling, increased release of inflammatory factors and oxidative damage, and elevated lung cell apoptosis. However, the rhKGF-2 intervention significantly improved the above pathological injuries, indicating that rhKGF-2 prevents severe SILI.

PI3K/Akt is an essential pathway regulating cell proliferation and apoptosis, which also contributes to acute lung injury. For example, Liang et al. showed that dexmedetomidine activates PI3K/Akt and dampens the expression of pro-inflammatory factors and facilitates the activity of SOD and CAT, thus alleviating ischemia/perfusion-induced severe lung injury (Liang et al., 2019). Moreover, the PI3K/Akt pathway indirectly activates the reactive oxygen species. A high concentration of reactive oxygen species oxidizes the cysteine residues on Keap1, thereby translocating Nrf2 to the nucleus and inducing the expression of genes containing antioxidant response elements, which helps maintain the redox balance (Koundouros and Poulogiannis, 2018). Nrf2 is a key regulator of redox homeostasis and plays a beneficial role in the lungs against oxidative stress. For example, Crocin prevents oxidative stress by activating Nrf2, thereby attenuating cigarette smoke-induced lung injury (Dianat et al., 2018). Xanthohumol activates the AMPK/GSK3β-Nrf2 axis and dampens the Txnip/NLRP3 inflammasome and NF-κB, which reduces oxidative stress and ameliorates LPS-induced acute lung injury (Lv et al., 2017). In addition, Ulinastatin inhibits JNK/NF-κB and activates PI3K/Akt/Nrf2 to reduce the pro-inflammatory cytokines and oxidative damage, thereby preventing LPS-induced mouse macrophage RAW264.7 inflammation (Li et al., 2018). Chen et al. confirmed that Ginsenoside Rb1 attenuates inflammation and oxidative stress by activating PI3K/Akt/Nrf2, thus alleviating ischemia/reperfusion-induced injury (Chen et al., 2019). It is worth noting that the activation of Nrf2 by PI3K/Akt is key to prevent hyperoxia-induced acute lung injury (Reddy et al., 2015). Fortunately, our results also illustrated that activating PI3K/Akt/Nrf2 protects the lungs and significantly abates the smoke inhalation-induced inflammation and oxidative damage. Meanwhile, the use of the PI3K inhibitor demonstrated that the PI3K/Akt/Nrf2 activation is critical for preventing SILI.

FoxO1 is a transcription factor with rich functions. In addition to regulating oxidative stress, immune homeostasis, cell apoptosis, etc., it also directly regulates inflammation through signal transduction, transcription regulation, or combination with other transcription factors (Sundaresan and Puthanveetil, 2017). For example, Schisandrin has been shown to ease inflammation by inhibiting the TLR-4/NF-κB/MAPK and Akt/FoxO1 signaling pathways, thus alleviating LPS-induced lung injury (Sun et al., 2018). More importantly, Liu et al. demonstrated that down-regulation of CD28 ameliorates inflammation, oxidative damage and blast exposure-induced lung injury by activating p-PI3K and p-Akt and inhibiting p-FoxO1 (Liu et al., 2019d). FoxO1 was found to inactivate NLRP3 inflammasome and thus down-regulate IL-1 (Kim et al., 2017). NLRP3 inflammasome is activated in diversified lung injuries. For instance, Zhang et al., 2020; showed that Glycine prevents LPS-induced lung injury by inactivating NLRP3 and enhancing Nrf2 (Sun et al., 2018). Other scholars have found that Diosmetin attenuates LPS-induced acute lung injury by activating Nrf2 and inhibiting NLRP3 (Liu et al., 2018). Our study demonstrated that regulating the PI3K/Akt/FoxO1-NLRP3 axis affects SILI. Specifically, the activated PI3K/Akt pathway inhibits the FoxO1-NLRP3 axis, which reduces inflammation and oxidative stress on lung tissue, and prevents severe SILI.

In summary, rhKGF-2 prevents SILI by activating PI3K/Akt/Nrf2 and inhibiting FoxO1-NLRP3 inflammasome, thus reducing inflammation, oxidative stress, and apoptosis. However, several limitations were also remained. First, we only evaluated the functions of rhKGF-2 in lung epithelial cells and monocytes, while its role in other lung cells and immune cells was poorly explored. Second, this study only testified the efficacy of rhKGF-2 in the SILI animal model, and its effect on SILI patients should verified in the future. Therefore, the influence of rhKGF-2 on the survival of SILI patients should be further studied on the basis of previous experiments to explore the more detailed pathological mechanism.

## Data Availability

The raw data supporting the conclusions of this article will be made available by the authors, without undue reservation, to any qualified researcher.
